# Harnessing Endophytic Fungi as a Sustainable Source of Novel Anticancer Agents: Opportunities, Challenges, and Future Directions

**DOI:** 10.3390/molecules31040693

**Published:** 2026-02-17

**Authors:** Elly Lowen, Simon E. Moulton, Enzo A. Palombo, Faith Kwa, Bita Zaferanloo

**Affiliations:** 1 Department of Chemistry and Biotechnology, School of Science, Computing and Emerging Technologies, Swinburne University of Technology, Melbourne, VIC 3122, Australia; 2 Department of Electrical, Robotics and Biomedical Engineering, School of Engineering, Swinburne University of Technology, Melbourne, VIC 3122, Australia; smoulton@swin.edu.au; 3 Iverson Health Innovation Research Institute, Swinburne University of Technology, Melbourne, VIC 3122, Australia; 4 Aikenhead Centre for Medical Discovery, St Vincent’s Hospital Melbourne, Melbourne, VIC 3065, Australia; 5 Department of Advanced Components and Materials Engineering, Sunchon National University, 255 Jungang-ro, Suncheon-si 57922, Jellanam-do, Republic of Korea; 6 Department of Biomedical, Health and Exercise Science, Swinburne University of Technology, Hawthorn, VIC 3122, Australia; fkwa@swin.edu.au

**Keywords:** Endophytic fungi, secondary metabolites, anticancer drug discovery, apoptosis, oncogenic signaling, synthetic biology

## Abstract

Despite significant advances in oncology, current cancer therapies remain constrained by toxicity, resistance, and limited selectivity. Endophytic fungi symbiotic microorganisms inhabiting plant tissues represent a sustainable and underexplored source of structurally diverse anticancer metabolites. These include alkaloids, terpenoids, polyketides, and peptides that disrupt microtubule dynamics, interfere with DNA replication, and induce mitochondrial-mediated apoptosis. They also modulate key oncogenic signalling pathways such as nuclear factor kappa B (NF-κB), signal transducer and activator of transcription 3 (STAT3), and phosphatidylinositol-3-kinase/protein kinase B (PI3K/Akt), thereby enhancing the efficacy of existing chemotherapies. Endophyte derived compounds further inhibit angiogenesis, suppress metastasis, and stimulate immune responses, offering multi-target mechanisms with reduced toxicity. This review examines strategies that enhance the discovery and yield of these bioactive metabolites, including One Strain Many Compounds (OSMAC), microbial co-culture, epigenetic activation, genome mining, and synthetic biology. A comparative assessment of endophyte-derived versus conventional anticancer agents highlights their potential for scalable, eco-sustainable production. Collectively, endophytic fungi are positioned as promising contributors to the next generation of accessible, cost-effective, and environmentally responsible anticancer therapies.

## 1. Introduction

The World Health Organization says that cancer is responsible for one out of every six fatalities in the world [1]. Cancer remains a leading cause of global mortality, accounting for approximately one in six deaths worldwide [2]. Despite advances in surgery, chemotherapy, and radiotherapy, current treatment strategies are frequently limited by systemic toxicity, narrow therapeutic indices, tumor heterogeneity, and the emergence of multidrug resistance. These constraints underscore the urgent need for safer, more effective, and mechanistically diverse anticancer agents capable of overcoming resistance while minimizing harm to healthy tissues.

Natural products have historically played a central role in oncology, contributing directly or indirectly to a substantial proportion of approved anticancer drugs [2]. Compounds such as paclitaxel, vincristine, camptothecin, and podophyllotoxin exemplify the enduring clinical value of structurally complex, biologically evolved molecules. However, continued reliance on plant harvesting and complex chemical synthesis has raised concerns regarding sustainability, supply stability, and environmental impact [3].

Within this context, endophytic fungi microorganisms that reside asymptomatically within healthy plant tissues have emerged as a promising and underexplored resource for anticancer drug discovery [3,4]. Endophytes produce a wide array of secondary metabolites, including alkaloids, polyketides, terpenoids, peptides, and hybrid compounds, many of which exhibit potent cytotoxic, antiproliferative, and apoptosis-inducing activities [5]. Importantly, these metabolites frequently modulate multiple oncogenic pathways, offering mechanistic diversity that may reduce the likelihood of resistance development [6].

To date, only a small fraction of plant species has been investigated for their endophytic communities, indicating vast untapped biosynthetic potential [3]. Unlike plant-derived drugs, fungal endophytes are culturable, genetically adaptable, and amenable to fermentation-based production, positioning them as sustainable bio factories for anticancer agents [4].

### Scope and Aim of This Review Paper

This review focuses on plant-derived endophytic fungi as renewable sources of anticancer secondary metabolites. It first outlines the biological and ecological characteristics that underpin their biosynthetic diversity, then summarizes major classes of endophyte-derived anticancer compounds and their mechanisms of action. Finally, it examines translational strategies that enhance discovery, yield, and sustainability including OSMAC, co-culture, epigenetic activation, genome mining, and synthetic biology while highlighting current challenges and future directions.

## 2. Search Strategy and Selection Criteria

### 2.1. Review Design and Methodological Framework

This review was conducted using a structured literature selection framework informed by the PRISMA 2020 guidelines to comprehensively assess anticancer secondary metabolites derived from plant-associated endophytic fungi [7,8]. The methodological approach was designed to ensure transparency, reproducibility, and consistency with MDPI Molecules review standards, while accommodating the inherent heterogeneity of natural-product and pharmacological studies.

The scope of the review encompassed fungal secondary metabolites, their chemical classes, biological targets, mechanisms of anticancer action, and reported therapeutic outcomes, with particular emphasis on compounds evaluated in human or animal cancer models. Both standalone anticancer activities and adjuvant or chemo sensitizing effects were considered, reflecting growing interest in endophyte-derived metabolites as complementary agents in oncology.

### 2.2. Literature Search Strategy

#### 2.2.1. Data Sources and Search Period

A comprehensive literature search was performed across four major scientific databases: PubMed, Scopus, Web of Science and Google Scholar Citation management, cross-referencing, and citation expansion were supported using the Publish or Perish software (version 8.10). The search covered publications from January 2000 to August 2025, capturing both foundational discoveries and recent advances in endophytic fungal anticancer research.

#### 2.2.2. Search Terms and Query Design

Search queries were constructed using Boolean operators and database-specific syntax. Core keywords included:

“endophytic fungi”, “endophyte-derived metabolites”, “fungal secondary metabolites”, “anticancer”, “cytotoxicity”, “apoptosis”, “cell cycle arrest”, “mechanism of action”, “normal cell toxicity”, and “selectivity index”.

To capture studies aligned with modern discovery and dereplication workflows, supplementary terms such as “metabolomics”, “LC–MS/MS”, “molecular networking”, “genome mining”, and “biosynthetic gene clusters” were also applied where appropriate.

In addition, reference lists of key primary studies and authoritative reviews were manually screened to identify relevant publications not retrieved through database searches.

### 2.3. Study Screening and Eligibility Assessment

#### 2.3.1. PRISMA-Based Screening Process

The initial database search yielded 230 records. After removal of 45 duplicate entries, 189 unique records were screened based on title and abstract. During this phase, secondary literature (review articles, book chapters, and editorials) and studies not directly addressing the anticancer activity of endophyte-derived metabolites were excluded, resulting in the removal of 25 records.

**Figure 1:**
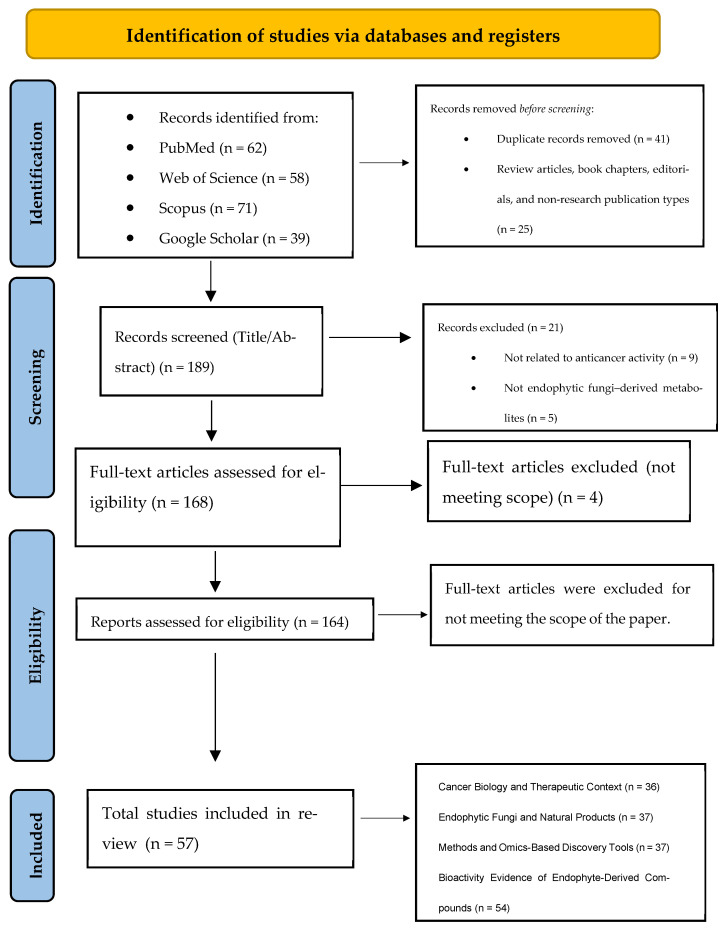
PRISMA 2020 flow diagram illustrates the identification, screening, eligibility assessment, and inclusion of studies evaluating anticancer secondary metabolites derived from plant-associated endophytic fungi. Adapted from [7] under CC BY 4.0.

A total of 164 full-text articles were subsequently assessed for eligibility. Following full-text evaluation, 160 primary studies met all inclusion criteria and were incorporated into the qualitative synthesis. The study selection process is summarized in the PRISMA 2020 flow diagram (Figure 1).

#### 2.3.2. Inclusion Criteria

- In vitro and/or in vivo evaluation of anticancer activity derived from endophytic fungal extracts, fractions, or purified metabolites;
- Use of human or animal cancer models with appropriate experimental controls;
- Reporting of quantitative cytotoxicity data, such as IC_50_ values or equivalent dose–response metrics;
- Inclusion of mechanistic validation, including but not limited to apoptosis induction, cell-cycle arrest, mitochondrial dysfunction, reactive oxygen species (ROS) generation, or modulation of oncogenic signaling pathways.

Where available, studies comparing cytotoxic effects in malignant versus non-malignant cells were prioritized to enable assessment of therapeutic selectivity.

#### 2.3.3. Exclusion Criteria

Studies were excluded if they:

- Focused exclusively on antimicrobial, agricultural, or ecological outcomes without anticancer evaluation;
- Lacked sufficient methodological detail or reproducible biological data;
- Failed to report quantitative cytotoxicity or mechanistic outcomes;
- Reported only preliminary screening observations without compound characterization or biological validation.

### 2.4. Data Extraction

Data from eligible studies were extracted using a standardized framework encompassing fungal taxonomy, host plant source, tissue niche, and ecological context, together with detailed information on metabolite class and chemical scaffold, including alkaloids, terpenoids, polyketides, peptides, and quinones. Extracted experimental parameters included cancer type, cell lines, animal models, and assay platforms used for biological evaluation, as well as quantitative bioactivity metrics such as IC_50_ values, dose–response behaviour, and selectivity profiles. Mechanistic information was recorded where available, including evidence for apoptosis induction, reactive oxygen species (ROS) modulation, mitochondrial dysfunction, cell-cycle perturbation, and pathway-level signalling changes. In addition, analytical and discovery approaches applied in each study such as dereplication strategies, metabolomics, molecular networking, genome mining, and biosynthetic gene cluster annotation were documented to support assessment of compound novelty and translational relevance.

### 2.5. Taxonomy-Based Data Categorization and Evidence Synthesis

To facilitate systematic comparison across heterogeneous experimental designs, included studies were organized using a taxonomy- and mechanism-based classification framework. Studies were categorized according to molecular targeting strategy (e.g., microtubules, topoisomerases, mitochondrial pathways, NF-κB, STAT3, PI3K/Akt), secondary metabolite class and composition, chemical structure and inferred biosynthetic origin, and mechanism of action with associated downstream cellular responses. Additional contextual variables, including cancer type, cellular model, assessment of toxicity toward normal cells, and safety-relevant outcomes, were also considered. Owing to substantial methodological heterogeneity across studies encompassing differences in biological models, assay platforms, exposure conditions, and reporting standards a qualitative synthesis approach was adopted rather than a quantitative meta-analysis. Findings were synthesized thematically according to metabolite class, fungal genus, and mechanistic pathway, enabling structured comparison while preserving biological and pharmacological nuance.

## 3. Endophytic Fungi and Their Relevance to Anticancer Drug Discovery

### 3.1. Definition and Biological Characteristics of Endophytic Fungi

Endophytic fungi are defined as fungi that colonize internal plant tissues without causing apparent disease symptoms throughout part or all of their life cycle [3]. They establish complex, often mutualistic interactions with their hosts, gaining access to nutrients and protective niches while contributing to host fitness through enhanced stress tolerance, pathogen resistance, and metabolic versatility [4]. From a biological perspective, endophytes are not defined by taxonomic rank but by lifestyle and function. They span diverse fungal lineages, predominantly within the Ascomycota, and exhibit extensive morphological and physiological plasticity [5]. This adaptability enables survival across heterogeneous plant tissues including roots, stems, leaves, bark, and seeds and under variable environmental conditions, which in turn shapes their secondary metabolic profiles.

### 3.2. Ecological and Evolutionary Drivers of Metabolite Diversity

The chemical diversity of endophytic fungi is closely linked to ecological and evolutionary pressures experienced by both the host plant and the associated microbiome. Environmental stressors such as drought, salinity, ultraviolet radiation, heavy metal exposure, and pathogen challenge select for endophytes capable of producing bioactive metabolites that mediate chemical defense and inter-organismal signaling [3,4]. These pressures promote diversification of biosynthetic gene clusters (BGCs) encoding polyketides, non-ribosomal peptides, terpenoids, and hybrid metabolites. Medicinal plants, endemic species, and hosts from extreme or underexplored habitats consistently yield endophytes with enhanced cytotoxic and antiproliferative activity [3]. This pattern reflects long-term co-evolution, in which chemically mediated interactions favor endophytes capable of producing metabolites with pharmacologically relevant scaffolds.

### 3.3. Biosynthesis Versus Bioconversion of Anticancer Metabolites

A critical issue in endophyte research is distinguishing de novo fungal biosynthesis from metabolite bioconversion. In several well-documented cases, endophytic fungi independently synthesize compounds structurally identical or analogous to host metabolites such as paclitaxel supported by the presence of complete fungal biosynthetic pathways and stable-isotope labeling evidence [5,9]. In other instances, endophytes enzymatically bioconvert host-derived precursors into structurally modified or more bioactive derivatives. Zaferanloo et al. [10] investigated endophytic fungi isolated from two indigenous Australian plants, *Eremophila longifolia* and *Eremophila maculata*. The fungal extracts demonstrated pronounced cytotoxic effects against human lung cancer cells as well as normal fibroblast lines, suggesting noteworthy anticancer potential but also highlighting the need for further evaluation of their selectivity and safety profiles.

Clarifying this distinction is essential for translational credibility. Contemporary best practices therefore include paired host–endophyte metabolomic analyses, stable-isotope labeling, and genome-based validation of biosynthetic plausibility. Together, these approaches reduce misattribution caused by host metabolite carryover and strengthen claims of fungal metabolic autonomy [9].

### 3.4. Sustainability and Biotechnological Advantages

Endophytic fungi offer several advantages over plant-based drug sourcing. They are fast-growing, cultivable under controlled conditions, and suitable for large-scale fermentation, enabling continuous production independent of season or geography [4]. Once isolated, strains can be cryopreserved and propagated indefinitely, reducing the need for repeated wild harvesting and supporting biodiversity conservation [3].

Moreover, fungal systems are amenable to strain improvement, metabolic engineering, and heterologous expression, allowing optimization of yield and production stability [11]. Collectively, these features position endophytic fungi as environmentally responsible and economically viable platforms for anticancer drug discovery and development.

#### Recent Research Highlights the Practical Impact of These Advantages

First, the cultivability of endophytic fungi has enabled reproducible access to bioactive metabolites without ecological depletion of host plants. Numerous studies summarized in Section 2.2 and Section 3 report successful isolation and fermentation of *Fusarium*, *Aspergillus*, *Cladosporium*, and *Penicillium* endophytes, yielding cytotoxic compounds such as paclitaxel, camptothecin, and beauvericin at laboratory scale [12,13,14,15,16]. This cultivability has facilitated repeated biological evaluation, mechanistic validation, and comparative pharmacological studies that would be impractical using slow-growing or endangered plant sources [13,17].

Second, fermentation scalability has supported measurable progress in yield optimization and preclinical development. As discussed in Section 4 and Section 5, advances in bioreactor cultivation, media optimization, elicitor application, and co-culture strategies have increased metabolite titers and production stability for compounds such as camptothecin, anthraquinone derivatives, and diverse polyketides [11,13,18]. These improvements have enabled in vivo validation in xenograft and zebrafish models and comparative efficacy studies against standard chemotherapeutics, representing a critical step beyond exploration in vitro screening [15,19].

Third, the genetic and epigenetic manipulability of endophytic fungi has driven a substantial expansion of chemical diversity. Recent applications of OSMAC strategies, epigenetic modifiers, genome mining, and synthetic biology approaches (Section 5) have successfully activated silent biosynthetic gene clusters, leading to the discovery of novel xanthones, indole derivatives, meroterpenoids, and polyketides with distinct anticancer mechanisms [20,21,22]. These outcomes underscore how endophytes function not only as sustainable producers of known anticancer agents but also as adaptable platforms for generating structurally and mechanistically novel lead compounds [13,23].

Collectively, these research advances demonstrate that the three advantages outlined above are not merely theoretical but have already translated into tangible progress in compound discovery, mechanistic understanding, and preclinical validation of endophyte-derived anticancer agents [13,15,17].

## 4. Mechanisms of Anticancer Action of Endophyte-Derived Compounds

Secondary metabolites from endophytes exhibit diverse and synergistic cytotoxic mechanisms that target key hallmarks of cancer. These compounds engage in multi-target actions, offering an advantage over traditional single-target chemotherapeutics. Their mechanisms include microtubule stabilization, topoisomerase inhibition, DNA intercalation, induction of ROS, and modulation of critical oncogenic signaling pathways such as NF-κB, STAT3, PI3K/Akt, and Mitogen-Activated Protein Kinase (MAPK). Certain compounds also enhance immune modulation and chemo sensitization, contributing to a comprehensive anticancer strategy.

### 4.1. Microtubule Disruption and Mitotic Arrest

#### 4.1.1. Paclitaxel

Paclitaxel (Taxol) is a clinically approved taxane diterpenoid and a cornerstone microtubule-stabilizing agent widely used in the treatment of solid tumors, including ovarian, lung, prostate, and Kaposi’s sarcoma [24,25]. In addition to its plant origin from *Taxus* spp., several endophytic fungi have been identified as alternative biosynthetic sources of paclitaxel and its precursors, including *Talaromyces andreanae*, *Seimatoantlerium nepalense*, *Alternaria alternata*, and *Chaetomella raphigera* [26,27]. Fungal-derived paclitaxel and baccatin III have demonstrated significant cytotoxicity and apoptosis induction across multiple cancer models, with reported efficacy against breast and leukemia cell lines at micromolar concentrations [28]. Mechanistically, paclitaxel stabilizes microtubules by preventing depolymerization, leading to G2/M cell-cycle arrest and apoptosis through modulation of Bax/Bcl-2 ratios [24,25].

Closely related microtubule- and DNA-targeting natural products include podophyllotoxin and its derivatives, which exhibit broad anticancer activity against leukemia, testicular, prostate, lung, and ovarian cancers [24]. Podophyllotoxin exerts its effects primarily through inhibition of topoisomerase II, resulting in DNA double-strand breaks and apoptotic cell death, while its semi-synthetic derivative etoposide enhances cytotoxicity by amplifying DNA damage and genomic instability [25]. Collectively, these findings highlight the potential of endophytic fungi as sustainable sources of clinically relevant microtubule- and DNA-targeting anticancer agents.

#### 4.1.2. Vinblastine

Vinblastine is a clinically approved vinca alkaloid anticancer drug that was first discovered in the late 1950s from the medicinal plant *Catharanthus roseus* [29,30]. These compounds are widely used in the treatment of hematological malignancies and solid tumors, including lymphoblastic leukemia and lymphoma.

In addition to their plant origin, vinblastine has been reported to be produced by endophytic fungi, including *Fusarium oxysporum* isolated from *Catharanthus roseus*, highlighting the potential of endophytes as alternative biosynthetic sources [31,32]. Fungal-derived vinblastine and related metabolites remain at the preclinical research stage, with ongoing efforts focused on strain identification, yield optimization, and biosynthetic pathway elucidation rather than clinical redeployment [13].

Endophytic fungal metabolites associated with vinblastine production have demonstrated notable cytotoxic activity against cancer cell lines, including lymphoblastic leukemia and HepG-2 (hepatocellular carcinoma) cells, with reported IC_50_ values as low as 7.48 µg/mL [23]. To contextualize this potency, conventional chemotherapeutic agents such as paclitaxel, doxorubicin, and 5-fluorouracil typically exhibit IC_50_ values in the micromolar range under comparable in vitro conditions [17,24]. These findings highlight the strong antiproliferative potential of endophyte-associated vinblastine and related extracts.

Mechanistically, vinblastine exerts its anticancer activity by binding to tubulin, thereby inhibiting microtubule polymerization, arresting cells at the metaphase–anaphase transition, and ultimately inducing apoptosis [33,34,35]. Fungal vinblastine has been shown to suppress the growth of CHO-K1, MCF-7, and HepG-2 cells [14]. Moreover, synergistic anticancer effects have been reported when vinblastine is combined with indirubin in MCF-7 cells or with paclitaxel and zerumbone in HeLa cells, further supporting its therapeutic relevance [15,18].

### 4.2. DNA Interaction and Topoisomerase Inhibition

#### 4.2.1. Camptothecin

Camptothecin is a clinically important natural product lead (quinoline alkaloid) that was first discovered in 1966 from the plant *Camptotheca acuminata* and later also identified in *Nothapodytes nimmoniana* [36,37]. While camptothecin itself is not widely used directly due to toxicity and solubility limitations, several semi-synthetic derivatives are clinically approved and in routine use as topoisomerase I inhibitors (e.g., irinotecan and topotecan) [38,39]. In recent years, endophytic fungi have been investigated as alternative and sustainable microbial sources of camptothecin, particularly endophytes associated with camptothecin-producing host plants [40]. Notably, camptothecin isolated from the endophytic fungus *Aspergillus flavus* exhibited pronounced antiproliferative activity against multiple human cancer cell lines, with the highest potency observed in hepatocellular carcinoma cells, and yield-enhancement strategies (e.g., methyl jasmonate elicitation and statistical optimization) further supported the feasibility of fungal production platforms at the preclinical/biotechnological development stage [41]. Additional reports describe camptothecin biosynthesis by endophytic *Aspergillus niger* associated with *Nothapodytes foetida*, with cytotoxic effects in liver, lung, ovarian, and colorectal cancer models [39]. Mechanistically, camptothecin exerts anticancer activity by stabilizing the Topoisomerase I–DNA cleavage complex, preventing DNA strand religation and inducing replication stress, cell-cycle arrest, and apoptosis through pathways involving p53, p21, mTOR, ERK, and Nrf2 signaling [38].

#### 4.2.2. Podophyllotoxin

Podophyllotoxin is a plant-derived aryltetralin lignan first isolated in the late 1940s from *Podophyllum* species, including *Podophyllum peltatum* and *Sinopodophyllum hexandrum* [42]. Although podophyllotoxin itself is not used clinically due to unacceptable toxicity, it serves as the structural precursor for the clinically approved, semi-synthetic anticancer drugs etoposide and teniposide, which are widely used in combination chemotherapy regimens for lung cancer, testicular cancer, lymphomas, and leukemia [24,25].

In parallel with plant-based production, endophytic fungi associated with podophyllotoxin-producing plants, including *Podophyllum peltatum*, *Sinopodophyllum hexandrum*, and *Dysosma veitchii*, have been reported to biosynthesize podophyllotoxin, positioning these microorganisms as alternative and sustainable production platforms at the preclinical/biotechnological research stage [42]. Mechanistically, podophyllotoxin and its derivatives exert anticancer activity primarily through topoisomerase II inhibition, resulting in impaired DNA replication, double-strand breaks, cell-cycle arrest, and apoptosis; etoposide enhances this effect by stabilizing the topoisomerase II–DNA cleavage complex, leading to extensive genomic damage and programmed cell death [25].

### 4.3. Mitochondrial Dysfunction and ROS Generation

#### 4.3.1. Beauvericin

Beauvericin is a fungal-derived cyclic hexadepsipeptide mycotoxin that was first reported in the 1970s and is produced by several filamentous fungi, including endophytic and entomopathogenic species of *Fusarium* and *Beauveria*. Beauvericin is not a commercial or clinical drug and is currently investigated as a preclinical anticancer lead.

Mechanistically, beauvericin exerts anticancer activity primarily through mitochondrial dysfunction and oxidative stress, leading to ROS-dependent apoptosis. In hepatocellular carcinoma, triple-negative breast cancer, and colorectal cancer models, beauvericin increases intracellular ROS, disrupts mitochondrial membrane potential, alters Bax/Bcl-2 ratios, activates caspase-9 and caspase-3, and modulates PI3K/Akt and MAPK signaling pathways [43]. These effects result in inhibition of tumor cell proliferation, suppression of epithelial–mesenchymal transition (EMT), and reduced migration in aggressive cancer cell lines, with additional anti-angiogenic activity observed at sub-lethal concentrations [44]. Beyond direct cytotoxicity, beauvericin has also been shown to activate dendritic cells via TLR4 signaling, indicating a potential immunomodulatory contribution to its anticancer effects [43]. Importantly, a recent EFSA scientific opinion concluded that beauvericin does not exhibit direct genotoxicity; observed DNA damage at high doses is secondary to ROS generation and mitochondrial stress, underscoring the need for careful dose-dependent toxicological evaluation in further preclinical development [45].

#### 4.3.2. Hypericin

Hypericin is a naturally occurring anthraquinone derivative first isolated in 1950 from the medicinal plant *Hypericum perforatum* [46,47]. Hypericin is not a commercially approved anticancer drug; however, it has progressed to the preclinical and early clinical-adjacent research stage, primarily in the context of photodynamic therapy (PDT) [48]. In addition to its plant origin, hypericin has been reported to be biosynthesized by endophytic fungi associated with *H. perforatum*, including *Thielavia subthermophila*, supporting the feasibility of microbial production platforms [13].

At the molecular level, hypericin exerts anticancer activity predominantly through ROS generation and mitochondrial dysfunction, leading to activation of intrinsic and extrinsic apoptotic pathways. Across multiple cancer models, including breast, colorectal, thyroid, skin carcinoma, leukemia, and T-cell lymphoma cell lines, hypericin has been shown to induce caspase-3 and caspase-8 activation, cytochrome c release, modulation of Bax/Bcl-2 ratios, and G2-phase cell-cycle arrest via regulation of p21, p27, and cyclins [49]. Its anticancer efficacy is particularly pronounced under photodynamic conditions, where improved oxygenation and advanced delivery systems enhance tumor cell susceptibility. Recent formulation and combination strategies, including nanocarrier-based delivery and synergistic drug combinations, have further improved tumor selectivity and reduced toxicity towards normal cells, positioning hypericin as a versatile, multi-target preclinical anticancer candidate, while highlighting the need for optimization of formulation and light-delivery parameters for future clinical translation.

### 4.4. Oncogenic Signaling Pathway Modulation

#### 4.4.1. Diosgenin

Diosgenin is a plant-derived steroidal sapogenin originally isolated from *Dioscorea* species and remains at the preclinical stage of anticancer development. It has demonstrated broad antiproliferative activity across multiple cancer models, including breast, lung, hepatic, prostate, colon cancer, and chronic myeloid leukemia [50].

Preclinical studies consistently show that diosgenin suppresses tumor growth, inhibits metastatic progression, and induces apoptosis. Significant tumor reduction has been reported in murine models of prostate and hepatic cancer following diosgenin administration, with subsequent studies confirming inhibitory effects in colon and breast cancer systems [51,52,53]. More recently, formulation strategies have enhanced their therapeutic efficacy; notably, diosgenin-loaded gold nanoparticles exhibited markedly increased cytotoxicity against colorectal (HCT116) and breast (HCC1954) cancer cell lines [54].

At the molecular level, diosgenin exerts anticancer effects through modulation of multiple oncogenic signaling pathways. These include suppression of NF-κB activation and osteoclast genesis [55], inhibition of AKT/mTOR/JNK signaling in HER2-positive breast cancer cells [14], and blockade of STAT3 signaling via JAK and SRC kinases in hepatocellular carcinoma models [56]. Generally, these findings position diosgenin as a multi-target preclinical anticancer lead with broad activity across tumor types and signaling networks.

#### 4.4.2. Toosendanin (TSN)

Toosendanin (TSN) is a plant-derived triterpenoid isolated from *Melia* species and remains at the preclinical stage of anticancer development. TSN and its derivative Isotoosendanin (ITSN) have demonstrated cytotoxic activity across a broad range of malignancies, including leukemia, colorectal, hepatic, prostate, gastric, breast, lymphoma, and glioma cancers [57].

Mechanistic studies indicate that TSN exerts anticancer effects through disruption of multiple oncogenic signaling pathways, notably PI3K/Akt, MEK/ERK, and MAPK/JNK, resulting in cell-cycle arrest and apoptosis [58]. In glioblastoma models, TSN inhibited proliferation of U87 and U6 cells via estrogen receptor-dependent mechanisms [59], while in gastric cancer systems it induced G1/S arrest and caspase-dependent apoptosis, likely mediated by activation of the p38 MAPK pathway [51].

Notably, TSN and ITSN have shown pronounced activity against triple-negative breast cancer, significantly reducing cell viability in MDA-MB-231, BT549, and 4T1 models through downregulation of pro-caspase-3 and Bcl-xL, leading to apoptosis, necrosis, and autophagy [60]. Additional studies have demonstrated that TSN suppresses glioma cell proliferation by inhibiting the PI3K/Akt/mTOR axis [61]. The bottom line is that these findings highlight TSN and ITSN as promising multitarget triterpenoid leads with broad anticancer potential warranting further preclinical development.

### 4.5. Recent Advances of New Endophyte-Derived Compounds and Their Mechanisms

Recent research has substantially broadened the chemical and biological landscape of endophyte-derived anticancer metabolites, reinforcing endophytic fungi as a persistent source of structurally diverse and biologically potent natural products. Most newly reported compounds remain at the preclinical stage, with evaluation centered on in vitro cytotoxicity, mechanism-based assays, and selective in vivo validation.

Early reports during this period described a range of bioactive metabolites, including resveratrol produced by the endophytic fungus *Quambalaria cyanescens* [13], xanthone derivatives such as griseophenexanthone A and digriseophene A from *Penicillium* species [62], and indole alkaloids isolated from *Curvularia geniculate* [61]. Additional findings included volatile organic compounds from *Penicillium oxalicum* with cytotoxic effects against breast and lung cancer cell lines [63], as well as fungal L-asparaginase from *Lasiodiplodia theobromae*, which exhibited selective activity against leukemia cells with reduced toxicity towards normal cells [41]. These metabolites were predominantly derived from plant-associated endophytes and commonly displayed low-micromolar IC_50_ values across multiple tumor models.

More recent studies demonstrate continued progress in both chemical novelty and translational relevance. Newly identified polyketides and meroterpenoids from endophytic *Fusarium*, *Aspergillus*, and *Cladosporium* species showed strong antiproliferative activity against breast, colorectal, lung, and glioma cell lines [51]. Importantly, several investigations incorporated comparative cytotoxicity assays using normal human fibroblasts or epithelial cells, reporting favorable selectivity indices [64]. In parallel, mangrove-derived endophytic fungi yielded novel anthraquinone and xanthone analogues that induced apoptosis through reactive oxygen species generation and mitochondrial dysfunction while maintaining reduced toxicity towards non-malignant cells [65].

Enzyme-based anticancer strategies have also gained momentum, with endophytic fungal L-asparaginase variants demonstrating enhanced efficacy against leukemia and breast cancer models alongside improved tolerability compared with bacterial counterparts [66]. Although none of these compounds have yet advanced to clinical trials, the growing emphasis on normal-cell toxicity assessment, mechanistic validation, and biosynthetic characterization reflects increasing translational maturity within the field.

Recent investigations have uncovered a growing range of endophyte-derived anticancer metabolites that extend well beyond classical scaffolds such as Taxol and camptothecin, highlighting the expanding chemical and biological diversity of these natural sources. Newly reported compounds demonstrate potent anticancer activity through diverse mechanisms, including apoptosis induction, mitochondrial dysfunction, ROS generation, and cell-cycle arrest. For example, resveratrol isolated from *Quambalaria cyanescens* markedly reduced A549 lung cancer cell viability through apoptosis induction and cell-cycle arrest [67]. Xanthone derivatives, including griseophenexanthone A and digriseophene A from *Penicillium* sp. ct-28, exerted cytotoxic effects against HepG2 liver cancer cells via cytochrome c–mediated apoptotic pathways [68]. An indole-based metabolite, 2-methyl-7-phenylindole from *Curvularia geniculata*, further illustrated mechanistic diversity by activating mitochondrial apoptotic signaling [69]. In addition, volatile organic compounds produced by *Penicillium oxalicum* were shown to suppress cancer cell proliferation and induce apoptosis [70], while L-asparaginase from *Lasiodiplodia theobromae* exhibited selective cytotoxicity against MCF-7 breast cancer cells through apoptotic signaling pathways [41].

**Figure 2:**
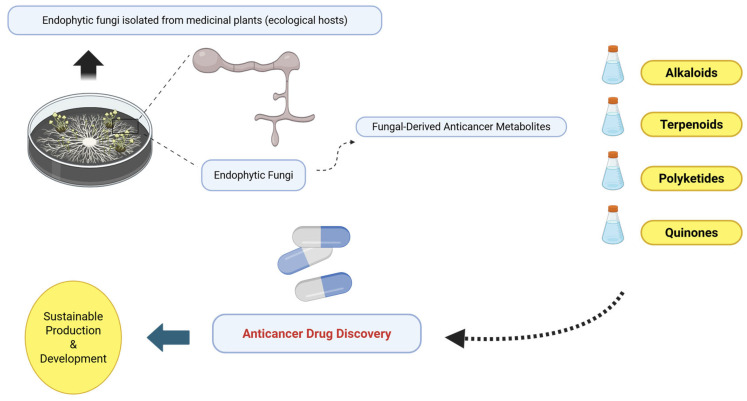
Conceptual overview illustrating endophytic fungi associated with medicinal plants as sources of anticancer metabolites. Medicinal plants (e.g., *Taxus*, *Camptotheca*, and *Catharanthus*) are shown exclusively as ecological hosts of endophytic fungi, while emphasis is placed on fungal-derived secondary metabolites including alkaloids, terpenoids, polyketides, and quinones and their relevance to anticancer drug discovery and sustainable production strategies. Created with BioRender.com on 19 January 2026.

**Figure 3:**
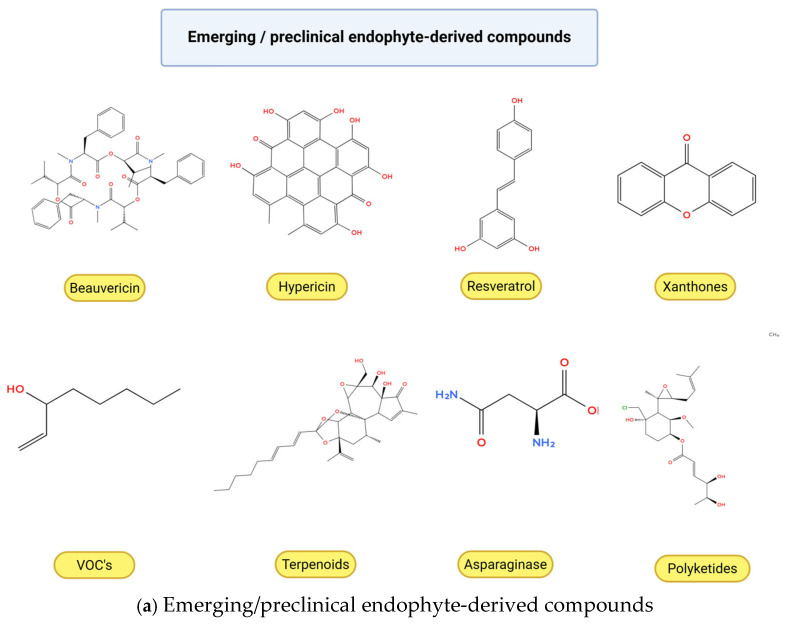

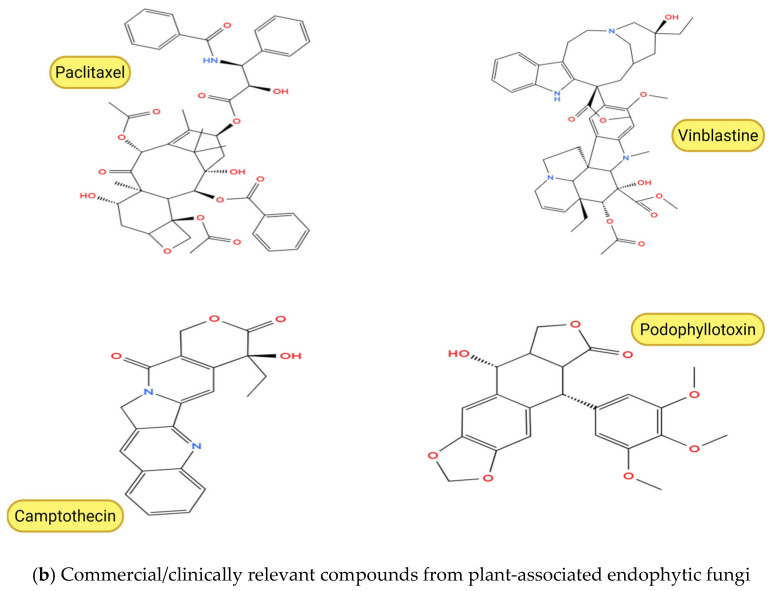
Chemical structures of anticancer compounds from plant-associated endophytic fungi. (**a**) Emerging and pre-clinical metabolites. (**b**) Clinically approved or commercially relevant agents and their core scaffolds. Created with BioRender.com, https://www.biorender.com/ (accessed on 10 February 2026). Oxygen atoms (O) and hydroxyl groups (–OH) are depicted in red in accordance with standard chemical structure conventions to facilitate visual identification of oxygen-containing functional groups.

Structural diversification was also evident in Taxol derivatives isolated from *Pseudodidymocyrtis lobariellae*, which displayed enhanced cytotoxic potential across multiple cancer cell lines [71]. Terpenoids derived from mangrove-associated endophytes of *Laguncularia racemosa* triggered mitochondrial apoptosis in human carcinoma cells [72], and the polyketide comoclathrin from *Comoclathris* sp. induced ROS-mediated apoptotic cell death, further expanding the repertoire of bioactive endophytic metabolites [73]. Figure 2 illustrates the core chemical scaffolds of these representative compounds, highlighting their substantial structural diversity. The chemical structures of representative emerging and clinically relevant compounds are presented in Figure 3. Overall, these findings confirm that endophytic fungi remain a dynamic and expanding reservoir of anticancer lead compounds. The combination of structural diversity, low-micromolar cytotoxic potency, and emerging selectivity profiles underscores their promise for future drug development and highlights the importance of integrated chemical, biological, and pharmacological evaluation in advancing endophyte-derived therapeutics.

### 4.6. Mechanistic Overview by Chemical Class

The information provided above enhances our understanding of the diverse anticancer pathways mediated by endophyte-derived secondary metabolites. These chemical classes encompass the major metabolites described above, establishing a clear connection between structural diversity and the wide range of anticancer mechanisms mediated by endophyte-derived compounds. The main compound classes such as alkaloids, terpenoids, polyketides, peptides, lignans, and quinones exert their effects through distinct mechanisms. Table 1 highlights key endophyte-derived metabolites with potent anticancer activity, their fungal sources, structural scaffolds, mechanisms of action, and target cancer cell lines. The listed compounds ranging from taxanes and alkaloids to terpenoids and polyketides illustrate the structural and mechanistic diversity underpinning the therapeutic potential of endophytic fungi.

## 5. Translational Insights and Pharmacological Validation

Recent advances in endophyte-based pharmacology have strengthened the translational bridge between laboratory discovery and therapeutic application by integrating in vitro screening, in vivo preclinical evaluation, comparative efficacy assessment, and formulation optimizations. Together, these complementary approaches enable a more rigorous evaluation of fungal metabolites, ensuring that promising compounds progress beyond preliminary cytotoxicity testing towards validated, scalable, and safe anticancer candidates suitable for further regulatory development [81,92,93,94].

### 5.1. In Vitro, Cytotoxicity and Mechanism-Based Assays

In vitro pharmacological assessments are essential for defining the anticancer potential of endophyte-derived metabolites. Cytotoxicity assays, including IC_50_ determination, NCI-60 profiling, and ROS measurement, establish potency and selectivity [95,96,97]. Mechanistic analyses based on caspase-3 activation, poly (ADP-ribose) polymerase (PARP) cleavage, cell-cycle arrest, and mitochondrial membrane depolarization provide insight into apoptotic and antiproliferative effects. Modulation of key signaling pathways such as NF-κB, STAT3, and p53/p21 further validates target engagement, while mechanisms including microtubule stabilization and topoisomerase inhibition highlight parallels with established chemotherapeutics [98,99,100]. Together, these integrated assays demonstrate the capacity of fungal metabolites to act on multiple cancer-relevant pathways, supporting their translational relevance.

### 5.2. In Vivo Pharmacological Studies and Preclinical Models

In vivo validation is a critical step in translational assessment and is commonly performed using mouse xenograft and zebrafish tumor models. These systems enable simultaneous evaluation of therapeutic efficacy, pharmacokinetic behaviors, and systemic safety, supported by readouts such as bioavailability, half-life, and tissue distribution [101,102]. Toxicological assessment further incorporates median lethal dose (LD_50_), hematological indices, and terminal deoxynucleotidyl transferase dUTP nick end labelling (TUNEL) assays to characterize tissue tolerance and apoptosis induction. Compounds that preferentially accumulate in tumor tissues while minimizing hepatic or renal toxicity demonstrate improved therapeutic indices relative to conventional agents. Collectively, these in vivo studies inform dose optimization and administration strategies and provide essential data to support progression towards Investigational New Drug (IND) applications [92,103].

### 5.3. Consideration of Cytotoxicity Toward Normal Cells and Therapeutic Selectivity

Given the well-documented systemic toxicity and dose-limiting adverse effects associated with conventional chemotherapeutic agents [104,105,106], particular emphasis was placed on studies assessing the selectivity of endophytic fungal extracts or metabolites toward malignant versus non-malignant cells. Where available, cytotoxicity data obtained from normal human cell models—including fibroblasts, epithelial cells, and peripheral blood mononuclear cells—were extracted and compared with cancer cell IC_50_ values to evaluate therapeutic selectivity [107,108]. Several investigations reported preferential toxicity toward cancer cells, supporting genuine anticancer selectivity, whereas others revealed non-selective cytotoxicity affecting both malignant and normal cells, underscoring the importance of incorporating normal-cell controls during early-stage screening [104,105,109]. For instance, metabolites isolated from endophytic fungi associated with Australian native plants exhibited potent activity against lung cancer cell lines but also affected normal fibroblasts, highlighting the necessity of selectivity profiling prior to translational consideration [107]. These findings reinforce the need to distinguish true anticancer activity from general cytotoxicity when evaluating the pharmacological relevance and clinical potential of endophyte-derived compounds [110,111].

### 5.4. Comparative Efficacy Against Standard Chemotherapies

In addition to assessing intrinsic cytotoxicity, preclinical models are increasingly employed to evaluate the ability of candidate compounds to overcome multidrug resistance (MDR), a major challenge in effective cancer chemotherapy. MDR most commonly arises from the overexpression of efflux transporters, such as P-glycoprotein, which reduce intracellular drug accumulation and consequently limit therapeutic efficacy. Resistant cancer cell lines and in vivo tumor models are therefore used to investigate whether natural products and their derivatives can modulate MDR-associated pathways, restore drug sensitivity, and enhance apoptotic responses. These studies typically integrate cytotoxicity assays, apoptosis analyses, and resistance-related biomarkers to comprehensively assess therapeutic potential while minimizing redundancy in experimental endpoints [112,113].

Beyond comparative efficacy against standard chemotherapeutics, progression of endophyte-derived compounds as MDR modulators requires early translational refinement. This includes rigorous dereplication and structural optimization to enhance potency, selectivity, and resistance-reversal capacity while minimizing off-target toxicity [17]. Safety pharmacology is particularly important for MDR-directed agents due to potential interactions with efflux transporters and associated signalling pathways [114]. In parallel, scalable fermentation must be established to ensure reproducible compound supply [10,115]. Formulation strategy further influences MDR performance: although dimethyl sulfoxide and ethanol facilitate compound dissolution, they may cause irritation or neurotoxicity at higher concentrations, whereas polyethylene glycol (PEG-400) and lipid-based carriers improve stability, intracellular delivery, and biocompatibility at increased cost [116].

### 5.5. From Lead Discovery to Drug Candidacy

Progression from lead discovery to drug candidacy requires a structured, multi-step refinement process encompassing dereplication, structural optimization, safety pharmacology, and scalable fermentation to ensure reproducible compound supply [6,103,117]. Bioreactor optimization, nutritional modulation, and elicitor-based strategies are commonly employed to enhance metabolite titers and address scale-up challenges. Formulation development is equally critical for balancing solubility, stability, bioavailability, and tolerability. While solvents such as dimethyl sulfoxide (DMSO) and ethanol facilitate compound dissolution, their use at higher concentrations may cause irritation or neurotoxicity. In contrast PEG-400 and lipid-based delivery systems improve distribution efficiency, stability, and biocompatibility, albeit at increased cost [118,119,120,121]. Collectively, these optimization strategies enhance formulation consistency, reduce toxicity, and support the transition of endophyte-derived metabolites towards clinical evaluation. The translational pharmacological characteristics, comparative efficacy, formulation considerations, and advancement criteria of representative endophyte-derived anticancer metabolites are summarized in Table 2.

### 5.6. Challenges and Opportunities for Clinical Advancement

Despite substantial progress, several bottlenecks continue to limit the clinical translation of endophyte-derived pharmaceuticals, including inconsistent metabolite yields, limited aqueous stability, and variable biosynthetic titers that hinder reproducibility and large-scale production [5,110,117,128,129]. Addressing these challenges requires the integration of metabolic engineering, heterologous gene expression, and synthetic biology approaches to stabilize biosynthetic pathways and enhance production robustness. In parallel, liposomal and nanoparticle-based encapsulation strategies are gaining traction, improving solubility, targeted delivery, and systemic tolerability. Equally important is early alignment of Good Manufacturing Practice (GMP) requirements to ensure quality control and facilitate regulatory progression from discovery to clinical evaluation [130,131,132]. Collectively, the convergence of advanced formulation technologies, scalable biosynthesis, and regulatory compliance is expected to accelerate the development of clinically viable endophyte-derived anticancer agents. Table 2 summarizes the pharmacological profiles, mechanisms of action, comparative efficacy, formulation considerations, and validated references for key endophyte-derived metabolites currently under pre-clinical and translational investigation.

## 6. Strategies for Enhancing Discovery and Sustainable Production of Antitumor Natural Products from Plant-Derived Endophytic Fungi

### 6.1. Targeted Bioprospecting and Habitat-Informed Fungal Selection

Targeted bioprospecting guided by ecological and habitat-specific considerations has become a decisive strategy for enhancing the discovery of antitumor metabolites from plant-derived endophytic fungi. Recent advances in fungal metabolomics demonstrate a strong correlation between ecological diversity and chemical diversity, indicating that environmental pressures play a central role in shaping secondary metabolite biosynthesis. Endophytic fungi inhabiting extreme or specialized environments such as mangrove ecosystems, alpine and desert plants, marine algae, and deep forest medicinal flora are subjected to constraints including salinity, drought, ultraviolet radiation, temperature extremes, heavy metal exposure, and pathogen pressure. These selective forces drive metabolic adaptation and frequently result in unique biosynthetic profiles with high pharmacological value [3,4].

Host plant identity and tissue niche (root, stem, leaf, bark, or seed) further influence endophytic community structure and metabolic output. Medicinal plants traditionally used for cancer, inflammation, or chronic disease management are consistently enriched in endophytes capable of producing cytotoxic, antiproliferative, and apoptosis-modulating metabolites, reflecting long-term coevolutionary selection for chemically mediated defense and signaling. Comparative bioprospecting studies report significantly higher bioactivity hit rates when prioritizing medicinal plants, endemic or extremophile species, and phylogenetically underexplored plant lineages, compared with random isolation strategies [3,4,13]. These endophytes frequently harbor biosynthetic gene clusters encoding structurally diverse polyketides, alkaloids, terpenoids, non-ribosomal peptides, and hybrid metabolites relevant to anticancer pharmacology.

Habitat-informed fungal selection has proven particularly productive for genera such as Fusarium, Cladosporium, Aspergillus, and Penicillium isolated from ecologically specialized niches, which have yielded structurally distinct naphthoquinones, anthraquinones, benzophenones, and terpenoids with potent cytotoxic activity [12,15]. These findings underscore the strong ecological influence on chemical novelty and reinforce the importance of continued bioprospecting in extreme and underexplored environments for identifying new anticancer lead compounds.

Effective “endophyte-first” discovery pipelines integrate methodological rigor to maximize novelty and reproducibility. Stringent surface sterilization protocols, sterility controls, and tissue-resolved isolation are essential to confirm true endophytic origin and to capture niche-specific metabolic diversity. Early-stage dereplication using morphological assessment, ITS/LSU sequencing, and genome-level fingerprinting reduces redundancy and prevents repeated cultivation of closely related isolates [5]. In parallel, front-loaded bioactivity screening using cancer-relevant phenotypes including tumor-selective cytotoxicity, apoptosis induction, reactive oxygen species modulation, and cell-cycle arrest enables prioritization of strains with mechanism-driven anticancer potential rather than nonspecific toxicity [4,5].

Given the continuing debate surrounding reports of “plant-like” metabolites from endophytes, contemporary discovery frameworks increasingly emphasize matched host–endophyte metabolomics, stable-isotope labelling, and genome-informed biosynthetic plausibility assessments. These integrated approaches minimize false attribution arising from host metabolite carryover, strengthen evidence for genuine fungal biosynthesis, and improve confidence in downstream pharmacological interpretation and translational relevance [5,9].

### 6.2. Awakening Silent Biosynthetic Gene Clusters

A substantial portion of fungal biosynthetic gene clusters (BGCs) remain transcriptionally silent under standard laboratory conditions, masking the full biosynthetic potential of endophytic fungi. Several strategies have been developed to activate these dormant clusters, including the OSMAC approach, co-culture with microbial competitors, and epigenetic modulation using compounds such as 5-azacytidine and suberoylanilide hydroxamic acid (SAHA). These treatments alter chromatin accessibility and activate stress-related signaling pathways, thereby uncovering new secondary metabolites such as anthraquinones, alkaloids, and phenolic dimers [133,134,135]. Successful examples include *Fusarium solani*, *Cladosporium* sp., and *Penicillium chrysogenum*, where these methods have yielded previously undetected bioactive scaffolds, including quisetin-like tetramic acids, yanuthone-class meroterpenoids, and penilactones [115,136]. Strain-specific activation strategies continue to be pivotal for unlocking the hidden chemical repertoire of fungal genomes.

### 6.3. Culture-Based Elicitation: OSMAC Designs, Co-Culture Systems, and Epigenetic Activation

A major limitation in endophyte-derived natural-product discovery is that many fungal BGCs remain silent or weakly expressed under standard laboratory monoculture conditions. Consequently, culture-based elicitation strategies remain essential for expanding accessible chemical space and revealing antitumor scaffolds [137,138]. The OSMAC paradigm is a cornerstone approach, systematically varying nutritional and physicochemical parameters including carbon and nitrogen sources, C:N ratios, pH, salinity, aeration, temperature, light exposure, and trace elements to perturb regulatory networks and activate cryptic biosynthesis.

Factorial and multivariate OSMAC designs outperform one-variable-at-a-time experiments by more efficiently sampling induction landscapes and improving reproducibility across laboratories [137]. When combined with LC–MS-based metabolomic profiling, OSMAC enables rapid identification of culture conditions that yield novel or significantly upregulated metabolites with anticancer potential.

Co-culture-based elicitation represents a powerful extension of OSMAC for plant-derived endophytes, as it more closely reflects the competitive and signaling interactions experienced in planta. Pairing endophytes with other fungi, bacteria, or host-associated microorganisms has repeatedly induced de novo metabolite production, increased titers of low-abundance compounds, and generated analog series with improved cytotoxic or apoptosis-inducing activity [138,139]. To improve reproducibility, recent frameworks emphasize controlled interaction modes such as contact-dependent versus diffusible signaling using partitioned systems, membrane inserts, staged inoculation, and time-resolved sampling [138]. Spatial metabolomics and imaging mass spectrometry further enable localization of induction zones, enhancing mechanistic interpretability [139].

Complementary to OSMAC and co-culture, chemical epigenetic elicitation has emerged as a scalable method to derepress secondary metabolism. Small-molecule modifiers targeting chromatin-associated processes, including histone deacetylase (HDAC) and DNA methyltransferase (DNMT) inhibitors, can activate silent BGCs and reveal otherwise inaccessible metabolite classes relevant to anticancer discovery [137,140]. Because epigenetic treatments may also amplify known metabolites, their application benefits from metabolomics-guided dereplication to minimize rediscovery.

Collectively, culture-based elicitation is most effective when implemented as a closed-loop workflow: experimental design → elicitation → LC–MS/MS profiling → dereplication and molecular networking → targeted isolation → bioactivity and mechanism-of-action evaluation → iterative refinement, improving both novelty rates and translational relevance [138,141].

### 6.4. Multi-Omics and Analytical Dereplication Tools

Integration of metabolomic, genomic, and transcriptomic platforms has revolutionized fungal natural product discovery by enabling rapid dereplication and compound validation. Analytical tools such as LC-MS/MS, HR-MS, NMR, and GNPS molecular networking allow for the differentiation between known and novel metabolites, minimizing redundancy in compound isolation. Parallel genome mining through antiSMASH, PRISM, and BiG-SCAPE software enables correlation of metabolite data with their respective BGCs, providing predictive insights into compound classes and regulatory mechanisms. Such multi-omics-guided dereplication has been successfully employed in *Fusarium*, *Aspergillus*, and *Cladosporium* species, linking Polyketide Synthase-Non-Ribosomal Peptide Synthetase (PKS–NRPS) hybrid gene clusters to several anticancer compounds [115]. Together, these analytical approaches streamline discovery pipelines and enhance accuracy in identifying promising fungal metabolites.

### 6.5. Omics-Driven Discovery and Dereplication: LC–MS/MS Molecular Networking, Paired Genomics–Metabolomics, and BGC Annotation

Current standards for rigor in natural-product research increasingly require clear demonstration of what metabolites are produced, why their biosynthesis is genetically plausible, and how novelty is established. This requirement is particularly critical in antitumor discovery, where rediscovery of known cytotoxins remains common [9,141].

As a result, omics-driven strategies integrating metabolomics, genomics, and bioinformatics have become central to endophyte-based discovery pipelines.

High-resolution LC–MS/MS metabolomics coupled with feature-based molecular networking enables rapid visualization of chemical families, detection of condition-specific metabolites, and prioritization of unique features lacking database matches. Community platforms and curated spectral libraries support early dereplication, allowing isolation efforts to focus on genuinely novel scaffolds [6,142,143]. Application of molecular networking across OSMAC and co-culture experiments further reveals inducible metabolite clusters that would otherwise remain undetected.

Integration of metabolomics with genome sequencing and BGC annotation provides an additional validation layer. Advances in fungal genome assembly and BGC prediction allow rapid identification of PKS, NRPS, terpene, and hybrid clusters in endophytic genomes. Correlating induced metabolite families with candidate BGCs using mass-shift logic, predicted tailoring enzymes, and expression patterns strengthens biosynthetic attribution and supports rational prioritization for pathway elucidation [141]. These paired genomics–metabolomics approaches also help distinguish truly novel metabolites from cryptic variants of known scaffolds.

Beyond discovery, omics-driven dereplication directly supports sustainability and scalability. Early identification of biosynthetic pathways enables promising antitumor metabolites to be routed toward strain improvement, heterologous expression, or synthetic-biology-based optimization, reducing reliance on repeated wild sampling and improving long-term supply prospects [9,141].

### 6.6. Genome Mining, Pathway Activation, and Synthetic Biology for Scalable Supply

Endophytic fungi typically encode far greater biosynthetic potential than is expressed under conventional cultivation, making genome mining and pathway engineering central to modern antitumor discovery. Multi-locus phylogenetics and whole-genome sequencing frequently reveal dense repertoires of BGCs whose products are absent from baseline metabolomes, providing a rational basis for targeted activation [115,135,144,145].

Genome mining is most informative when integrated with metabolomics, enabling identification of “expression gaps” and prioritization of clusters likely to yield drug-like scaffolds. Among activation strategies, CRISPR/Cas-based technologies have expanded functional genetics in non-model fungi. CRISPR-mediated transcriptional activation (CRISPRa) enables reversible induction of silent BGCs without permanent genome modification, facilitating systematic activation screens [92,146,147].

For sustainable supply, heterologous expression platforms are increasingly important. Many endophytes are slow-growing or genetically intractable; transferring validated BGCs into optimized fungal hosts, particularly Aspergillus chassis strains with reduced background metabolism, improves yield, reproducibility, and scalability [11,148]. Together, genome mining and synthetic biology support a dual model in which wild endophytes serve as discovery reservoirs and engineered platforms enable production.

### 6.7. Bioprocess Development for Sustainable Production: Fermentation Mode, Scale-Up, and Downstream Recovery

Translation of endophyte-derived metabolites frequently fails due to insufficient or inconsistent production, necessitating a bioprocess-centered framework encompassing strain stability, fermentation strategy, scale-up, and downstream recovery. Submerged fermentation offers scalability and control, while solid-state or hybrid systems may better support secondary metabolism in some endophytes [137,149].

Scale-up introduces predictable constraints, including oxygen transfer limitations, mixing heterogeneity, and morphological drift. Established frameworks emphasize scale-down modeling and oxygen transfer matching to preserve production-associated metabolic states [150]. Where co-culture systems are impractical at scale, defined elicitors or conditioned media components may partially substitute for microbial interactions [138].

Downstream recovery is equally critical, as many fungal metabolites partition into biomass or bind media components. Early assessment of metabolite distribution and integration of adsorbent resins or in situ extraction systems can stabilize low-abundance compounds and simplify purification [137]. Sustainable production ultimately requires reproducible, quality-controlled processes rather than maximal yield alone.

### 6.8. Translational Rigor, Sustainability, and Governance: From Credible Attribution to Responsible Bioprospecting

Translational value of endophyte-derived antitumor metabolites depends on three interdependent pillars: credible biosynthetic attribution, pharmacological rigor beyond nonspecific cytotoxicity, and responsible governance. Best practices increasingly emphasize paired host–endophyte metabolomics, stable-isotope labeling, and genome-informed plausibility assessments to strengthen biosynthetic claims [5,9].

Pharmacologically, early selectivity profiling against non-malignant cells, orthogonal viability assays, apoptosis and cell-cycle biomarkers, and preliminary mechanism-of-action hypotheses reduce over-prioritization of broadly toxic compounds [6,141].

Bioprospecting is also shaped by access and benefit-sharing requirements under the Nagoya Protocol, which governs use of genetic resources and downstream commercialization. Early integration of legal and ethical compliance mitigates translational barriers and aligns discovery with biodiversity stewardship [151]. When combined with scalable microbial production, endophyte-based pipelines offer conservation-positive alternatives to harvesting slow-growing medicinal plants, reinforcing the alignment between scientific rigor and sustainability [152,153,154].

## 7. Sustainability, Bioprospecting, and Future Perspectives

### 7.1. Persistent Research Gaps in Endophyte-Based Drug Development

Despite substantial advances, several limitations continue to constrain the full exploitation of endophytic fungi in anticancer drug development. Yield optimization remains a critical bottleneck, as existing cultivation systems often fail to deliver stable, reproducible, or scalable metabolite production. Although innovations such as Melanocyte Growth Medium (MGM) have enhanced fungal biomass accumulation and metabolite recovery, further optimization is required to improve production efficiency and compound purity [155]. In parallel, mechanistic understanding of the cytotoxic and apoptotic pathways triggered by fungal metabolites remains incomplete, limiting opportunities for rational drug design and target-based optimization [156,157]. Functional genomics and gene characterization studies are still relatively limited, and deeper insight into biosynthetic gene clusters and their regulatory architecture is essential to enable precise metabolic engineering and pathway activation strategies [23,158].

### 7.2. Genomics and Metabolomics Integration for Precision Discovery

The integration of genomic, transcriptomic, and metabolomic platforms has emerged as a powerful strategy for improving the precision and efficiency of endophyte-based drug discovery. Omics-driven approaches enable accurate strain prioritization, reveal cryptic biosynthetic potential, and establish direct links between secondary metabolites and their underlying biosynthetic gene clusters (BGCs), thereby accelerating compound validation and dereplication [142]. Comparative genomics and metabolite–gene association analyses facilitate the identification of previously overlooked or poorly expressed pathways that are often inaccessible through conventional culture-based methods. Furthermore, coupling transcriptomic profiling with metabolomic data allows dynamic gene expression changes to be correlated with metabolite production across varying environmental or co-culture conditions, strengthening functional assignment of bioactive compounds. In aggregate, these integrated platforms enhance screening accuracy, streamline discovery pipelines, and uncover multifunctional bioactivities including anticancer, antimicrobial, and antioxidant effects while providing deeper insight into endophyte–host interactions and metabolic adaptability [9,159].

### 7.3. Biodiversity-Driven Bioprospecting and Translational Challenges in Endophytic Fungal Anticancer Research

#### 7.3.1. Biodiversity-Driven Bioprospecting and Chemical Innovation

Sustained bioprospecting across diverse and underexplored ecological niches remains fundamental to expanding the chemical space of endophyte-derived anticancer metabolites. Endophytic fungi isolated from mangroves, alpine forests, arid deserts, and marine ecosystems consistently display pronounced metabolic plasticity, yielding structurally distinctive secondary metabolites with potent bioactivities [160]. These environments impose selective pressures such as salinity, temperature extremes, ultraviolet radiation, and nutrient limitation that promote adaptive secondary metabolism and favor the evolution of unique biosynthetic pathways.

Integrating biodiversity research with ecological metadata, environmental sequencing, and strain engineering has significantly enhanced the efficiency of fungal drug discovery. Such approaches enable predictive strain selection, reduce redundancy, and facilitate sustainable utilization of microbial resources. Biodiversity-centered exploration, when combined with omics-based selection strategies and synthetic biology, directly links ecological diversity to chemical novelty and translational innovation in anticancer drug discovery.

#### 7.3.2. Translational and Manufacturing Challenges

Despite the growing number of antitumor compounds reported from plant-derived endophytic fungi, their progression towards clinically relevant therapeutics remains constrained by persistent translational, manufacturing, and regulatory challenges. A primary limitation is the low, unstable, and often irreproducible production of bioactive secondary metabolites. This variability largely reflects the conditional expression of biosynthetic gene clusters (BGCs), many of which remain transcriptionally silent or weakly expressed under standard laboratory conditions [3]. Such instability complicates scale-up, restricts material availability for advanced pharmacological testing, and undermines reproducibility across studies.

Additional bottlenecks arise during compound isolation, purification, and dereplication. Although advances in chromatographic, spectroscopic, and metabolomics-based platforms have improved detection and structural characterization, the frequent rediscovery of known metabolites continues to consume significant time and resources [142,161]. These challenges are particularly pronounced when working with sterile mycelial endophytes, which lack diagnostic reproductive structures, complicating taxonomic identification and increasing susceptibility to contamination during prolonged cultivation and extraction [3].

From a translational perspective, insufficient evaluation of pharmacokinetics, toxicity, and in vivo efficacy represents a major barrier. While in vitro cytotoxicity and mechanistic studies dominate literature, comparatively few investigations advance to standardized animal models or systematically assess absorption, distribution, metabolism, and safety profiles [2,17,162]. This imbalance weakens cross-study comparability and limits the predictive value of early-stage screening for clinical relevance.

Future progress will require a decisive shift from discovery-centric workflows towards translation-ready development pipelines. Priority directions include:

(i) Synthetic biology and metabolic engineering to stabilize BGC expression and enable scalable, GMP-compatible production;
(ii) Multi-omics-guided dereplication and genome mining to accelerate lead prioritization and strengthen mechanistic validation;
(iii) Early integration of formulation science, predictive toxicology, and pharmacokinetic/pharmacodynamic modelling to align discovery outputs with regulatory expectations [163,164].

In summary, the absence of clinically approved endophyte-derived anticancer drugs reflects translational and manufacturing limitations rather than biological inadequacy. Addressing yield instability, host dependency, and late-stage pharmacological validation through integrated, multidisciplinary strategies will be essential to convert the substantial in vitro promise of fungal endophytes into clinically impactful anticancer therapies.

## 8. Conclusions

Endophytic fungi have emerged as promising and renewable sources of structurally diverse bioactive metabolites, exemplified by the pioneering discovery of Taxol from *Taxomyces andreanae.* These microorganisms offer a sustainable alternative to plant-based compound extraction, addressing issues of ecological strain and limited supply. Their ability to biosynthesize potent anticancer agents such as paclitaxel, camptothecin, podophyllotoxin, vinblastine, hypericin, diosgenin, and various polyketides underscores their chemical and pharmacological versatility. These metabolites not only serve as effective cytotoxic agents but also provide mechanistic insights into cancer biology, acting through pathways such as apoptosis induction, microtubule destabilization, and oxidative stress modulation. Despite this therapeutic promise, only a limited number of fungal metabolites have progressed into clinical development, highlighting a pressing need for further elucidation of their molecular targets, mechanisms of action, and biosynthetic pathways. Advancements in genomics, metabolomics, and synthetic biology now enable precise strain improvement, activation of silent biosynthetic gene clusters, and scalable production through engineered fermentation. Such integrative strategies can help overcome challenges related to low yields, host dependency, and translational bottlenecks. Moreover, endophytic fungi from underexplored habitats such as mangroves, arid zones, and high-biodiversity regions like Australia and Malaysia represent a largely untapped reservoir of novel pharmacophores. Their potential is further enhanced when considered in synergy with phytochemicals or conventional chemotherapeutics, offering avenues for combination therapies with improved efficacy and reduced toxicity. In conclusion, with continued bioprospecting, technological innovation, and multidisciplinary collaboration, endophytic fungi are poised to play a transformative role in the next generation of sustainable anticancer drug discovery and development.

## Figures and Tables

**Table 1:** Major anticancer compounds derived from plant-associated endophytic fungi and their principal mechanisms of action. All listed compounds were isolated from endophytic fungal sources or evaluated within the context of endophyte-associated natural products. Where IC_50_ values against normal human cells or assay-matched positive controls were not reported in the original studies, this is explicitly indicated to maintain data transparency. NR, not reported in the cited study. Chemical structures of all compounds are shown in Figure 2; in cases where normal-cell toxicity or matched positive controls were unavailable, selectivity indices could not be determined.

Compound	Endophytic Fungal Source(s)	Chemical Structure	Mechanism of Action	Target Cancer Type Cell Line(s)	Tumor Activity (IC_50_; Model)	Normal-Cell Toxicity (IC_50_; Model)	Development Status	Ref.
2-Methyl-7-phenylindole	*Curvularia geniculata*	Indole derivative	Activates mitochondrial apoptotic pathways	Not specified	Activates mitochondrial apoptotic pathways; tumor inhibition reported	NR	Preclinical	[74]
Asparaginase (L-asparaginase)	*Lasiodiplodia theobromae*	Protein enzyme	Induces apoptosis via L-asparagine depletion and signal activation	MCF-7 (breast)	Anti-leukemic activity reported; IC_50_ ~35.2 ± 0.7 U/mL (M-NFS-60)	CC_50_ ~79.4 ± 1.9 U/mL (WI-38)	Clinically used enzyme; endophytic source preclinical	[75]
Beauvericin	*Fusarium* spp., *Beauveria* spp.	Cyclic hexadepsipeptide	Induces ROS production; disrupts mitochondrial potential; activates caspase-9/3; PI3K/AKT and MAPK modulation	Hepatocellular-lar, MDA-MB-231, PC-3, Colo-rectal	Low µM range in breast, colorectal and prostate cancer models	Variable; NR in several studies	Preclinical	[44,64,76,77]
Camptothecin (CPT)	*Aspergillus flavus*, *A. niger*, *Nothapodytes foetida*	Quinoline alkaloid	Topoisomerase I; induces DNA damage and apoptosis via p53, mTOR, ERK pathways	HEPG-2, MCF-7, HCT-29, Ovarian, Lung	Low µM range against HepG2, MCF-7 and HCT-116 cells	NR	Clinically enabling lead (derivatives approved); fungal source: preclinical	[78,79,80,81]
Diosgenin	*Rhizopus oryzae*, *Fusarium* sp.	Steroidal sapogenin	Inhibits NF-κB, STAT3, AKT/mTOR pathways; reduces Skp2 and JNK signaling; induces apoptosis and anti-metastatic effects	Breast, Lung, Prostate, Colon, Hepatic carcinoma, CML	Low–mid µM across breast, prostate, colon and liver cancer models	NR	Preclinical	[82]
Hypericin	*Thielavia subther-mophila (from Hyper-icum perforatum)*	Anthraquinone derivative	Generates ROS; triggers caspase activation; releases cytochrome c; induces G2 arrest; enhances PDT response	T-cell lymphoma, Breast, Skin, Colorectal, Thyroid, Leukemia	Low µM; strongly PDT-dependent	Reduced toxicity under PDT; NR	Preclinical/clinical-adjacent (PDT)	[48]
Paclitaxel	*Talaromyces andreanae*, *Alternaria alternata*, *Seimatoantlerium nepalense*, *Chaetomella raphigera*	Taxane diterpenoid	Stabilizes microtubules; prevents de-polymerization; arrests cell cycle at G2–M phase; induces apoptosis via Bax/Bcl-2 modulation	MCF-7, HLK-210, HL-251, ovarian and hepatic carcinoma	nM–low µM range depending on cancer cell line	NR	Clinically approved drug; fungal production: preclinical/biotechnological	[48]
Podophyllotoxin	*Sinopodophyllum hexandrum*, *Dysos-ma veitchii*	Aryltetralin lignan	Topoisomerase II; induces double-strand DNA breaks; inhibits replication; triggers apoptosis (parent of etopo-side)	HL-60, NT2/D1, PC-3, A549, SKOV-3	Cytotoxic activity in leukemia, lung, breast and ovarian cancer models	NR	Preclinical lead; derivatives clinically approved	[24]
Polyketide “comoclathrin”	*Quambalaria cyanescens*	Polyketide	Induces ROS-mediated apoptosis; inhibits tumor growth	Multiple tumor cell lines	NR (no primary tumor IC_50_ located from accessible sources)	NR	Preclinical (insufficient primary quantitative data located)	[83]
Resveratrol	*Quambalaria cyanescens*	Stilbene polyphenol	Induces apoptosis; causes G1/S arrest; reduces A549 cell viability by 82%	A549 (lung)	~82% reduction in A549 viability; apoptosis + G1/S arrest reported	NR	Preclinical/nutraceutical-adjacent	[83,84]
Taxol derivatives	*Pseudodidymocyrtis lobariellae KL27*	Taxane diterpenoid variants	Microtubule stabilization and apoptosis	Multiple human cancers	Study demonstrates promotion of Taxol biosynthesis/accumulation in *Taxus*	NR	Preclinical/biotech (taxane production platform); parent drug approved	[85]
Terpenoids	*Laguncularia racemosa (mangrove endophyte) Mangrove plant Laguncularia racemosa as host origin reported in endophyte literature*	Isoprenoid terpenes	Induce mitochondrial apoptosis	Human carcinoma	NR in accessible sources for the specific “terpenoids” row (the host/endophyte link is documented, but quantitative tumor IC_50_ for this exact entry was not retrievable here)	NR	Preclinical (needs primary quantitative tumor data)	[72]
Toosendanin (TSN)	*Melia toosendan derived endophytes*	Triterpenoid li-monoid	Inhibits PI3K/Akt, MAPK, and JNK pathways; induces apoptosis via caspase activation; G1/S arrest	Leukemia, Colorectal, Hepatic, Prostate, Glioblastoma, TNBC	Low µM in glioma, gastric and TNBC models	NR	Preclinical	[85,86]
Vinblastine/Vincristine	*Catharanthus roseus*, *Fusarium oxysporum*	Vinca alkaloid	Binds tubulin; prevents microtubule polymerization; mitotic arrest (anaphase block); apoptosis	Lymphoblastic leukemia, HepG-2, MCF-7, CHO-K1, HeLa	As low as 7.48 µg/mL in selected in vitro assays	NR	Clinically approved drugs; fungal forms: preclinical	[87,88,89]
Volatile organic compounds (VOCs)	*Penicillium oxalicum*	Low-MW organics (mixed structure)	Induces apoptosis and suppresses proliferation	Not specified	Cytotoxic activity reported with IC_50_ values for breast cancer models including MDA-MB-231	NR	Preclinical	[90]
Xanthone derivatives	*Penicillium* sp. *ct-28*	Tricyclic aromatic xanthones	Cytochrome c–mediated apoptosis	HepG2 (liver)	Cytotoxicity against HepG2 reported for EtOAc extract and isolated xanthone/benzophenone constituents	NR	Preclinical	[91]

**Table 2:** Translational pharmacological profiles of representative endophyte-derived anticancer metabolites.

Compound/Class (Representative Source Endophyte)	Core Mechanism(s) of Action	In Vitro Cytotoxicity & Selectivity Profiling	In Vivo/Preclinical (Models, PK, Toxicology)	Comparative Performance vs. Conventional Chemotherapeutics	Criteria for Advancing Toward Drug Candidacy	Formulation Solvents & Implications	References
Vincristine/Vinblastine (*Catharanthus roseus* endophytes)	Disrupt mitotic spindle formation; inhibit microtubule assembly.	Potent activity across hematologic and solid tumor lines; mitotic arrest.	Validated in rodent xenografts; low systemic toxicity.	Comparable to clinical vinca alkaloids.	Reproducible yields; validated biomarkers.	DMSO/ethanol improve solubility but may induce neurotoxicity at high dose.	[122]
Camptothecin (CPT) (*Trichoderma atroviride*, *Nothapodytes* endophytes)	Topoisomerase I inhibition causes DNA replication block.	Strong activity in SW480, HepG2, A549 lines.	Validated in xenograft models; synergistic with mTOR inhibitors.	Comparable to Topotecan; reduced resistance.	Stable yield; confirmed topo-I engagement.	Formulated in PEG-400/DMSO mixtures; DMSO limited by injection-site irritation.	[40]
Paclitaxel (PTX) (*Taxomyces andreanae*, *Pestalotiopsis* spp.)	Microtubule stabilization; G2/M arrest; p53/p21 pathway activation.	High antiproliferative potency in A549, MCF-7.	Effective in breast and lung models.	Comparable or superior to standard PTX.	Scalable fermentation titers; toxicity assessed.	Lipid emulsions improve solubility; ethanol-based preps risk hypersensitivity.	[123]
Colchicine (*Diaporthe perseae*, *Cladosporium* spp.)	Inhibits microtubule polymerization; induces apoptosis.	Nanomolar IC_50_; tumor selective.	Preclinical tox data suggests narrow therapeutic window.	Like standard colchicine.	Validated biosynthesis; myelosuppression screening.	Ethanol and DMSO commonly used; solubility favorable but GI toxicity limiting.	[124]
Berberine (endophyte-linked)	Wnt/β-catenin inhibition; G1 arrest and apoptosis.	Potent in HCT116 and colon lines.	Colon xenografts validated; low systemic tox.	Synergistic with kinase inhibitors.	Optimized oral formulations under study.	Ethanol/water extracts used; moderate solubility and stability advantages.	[58]
Chaetominine (*Chaetomium* spp. IFB-E015)	Mitochondrial apoptosis via Bax/Bcl-2 modulation.	Nanomolar IC_50_; selective to leukemia lines.	Leukemia xenografts show strong PD activity.	Comparable to tubulin inhibitors.	Validated selectivity, tox screening.	DMSO and methanol-based extracts; stable but require cryoprotection.	[125]
Secalonic acid D (mangrove endophyte ZSU44)	GSK-3/β-catenin suppression; apoptosis induction.	Potent in leukemia lines.	Effective in hematologic xenografts.	Novel vs. topo/tubulin drugs.	Pathway biomarker validated.	PEG-400 and ethanol solubilizers enhance bioavailability; high-cost limits.	[31]
Ergoflavin (*Mimusops elengi* endophytes)	Suppresses TNF-α/IL-6; multi-target antiproliferative.	Micromolar IC_50_ in solid tumors.	Preclinical PK/tox ongoing.	Distinct scaffold from taxanes.	Cytokine modulation markers validated.	Ethanol and DMSO extracts; mild toxicity, stable in refrigerated storage.	[15]
Cladosporols (*Cladosporium cladosporioides*)	Disrupt mitochondrial membrane; regulate p21/cyclin D1.	Selective cytotoxicity in HeLa and colorectal lines.	Preliminary xenograft validation.	Comparable to microtubule inhibitors.	Tumor-specific and tolerable.	Methanol extracts yield stable formulations; moderate bioavailability.	[126]
L-Asparaginase (*Aspergillus* sp., *Cassia fistula* endophyte)	Depletes extracellular asparagine; inhibits protein synthesis.	Robust activity in leukemia lines.	Comparable to bacterial enzyme.	Parallel efficacy with reduced immunogenicity.	GLP tox and purity validation ongoing.	Aqueous/PEG systems preferred for enzyme stabilization; avoids DMSO toxicity.	[127]

## Data Availability

This article is a review and does not report original experimental data. All information analyzed is derived from previously published studies, which are cited in the reference list.

## Abbreviations

The following abbreviations are used in this manuscript:

AKT	Protein kinase B
BGC	Biosynthetic Gene Cluster
CML	Chronic myeloid leukaemia
CPT	Camptothecin
CRISPR/Cas9	Clustered Regularly Interspaced Short Palindromic Repeats/CRISPR-associated protein 9
DNMT	DNA methyltransferase
DMSO	Dimethyl sulfoxide
EFSA	European Food Safety Authority
EMT	Epithelial–mesenchymal transition
ERK	Extracellular signal-regulated kinase
GMP	Good Manufacturing Practice
GNPS	Global Natural Products Social Molecular Networking
GSK-3	Glycogen synthase kinase 3
HDAC	Histone deacetylase
HR-MS	High-resolution mass spectrometry
IC_50_	Half Maximal Inhibitory Concentration
IND	Investigational New Drug
ITS	Internal transcribed spacer
ITSN	Isotoosendanin
JAK	Janus kinase
JNK	c-Jun N-terminal kinase
LC–MS/MS	Liquid chromatography–tandem mass spectrometry
LD_50_	Median lethal dose
MAPK	Mitogen-Activated Protein Kinase
MDR	Multidrug resistance
MGM	Melanocyte Growth Medium
mTOR	Mechanistic target of rapamycin
NCI-60	National Cancer Institute 60 human tumour cell line panel
NF-κB	Nuclear Factor kappa B– Phosphatidylinositol 3-Kinase/Protein Kinase B
NMR	Nuclear magnetic resonance
NRPS	Non-ribosomal peptide synthetase
OSMAC	One Strain–Many Compounds
PARP	Poly (ADP-ribose) polymerase
PD	Pharmacodynamics
PEG-400	Polyethylene glycol 400
PDT	Photodynamic therapy
PI3K/Akt	Phosphatidylinositol-3-kinase
PK	Pharmacokinetics
PKS	Polyketide synthase
PKS–NRPS	Polyketide Synthase-Non-Ribosomal Peptide Synthetase
PRISMA	Preferred Reporting Items for Systematic Reviews and Meta-Analyses
ROS	Reactive Oxygen Species
SAHA	Suberoylanilide hydroxamic acid
STAT3	Signal Transducer and Activator of Transcription 3
TLR4	Toll-like receptor 4
TNBC	Triple-negative breast cancer
TSN	Toosendanin
TUNEL	Terminal deoxynucleotidyl transferase dUTP nick end labelling
VOCs	Volatile organic compounds
